# NLR- and *mlo*-Based Resistance Mechanisms against Powdery Mildew in *Cannabis sativa*

**DOI:** 10.3390/plants13010105

**Published:** 2023-12-29

**Authors:** Tiziana M. Sirangelo

**Affiliations:** ENEA-Italian National Agency for New Technologies, Energy and Sustainable Economic Development-Division Biotechnologies and Agroindustry, 00123 Rome, Italy; tiziana.sirangelo@enea.it

**Keywords:** *Cannabis sativa*, powdery mildew, mildew resistance locus o, nucleotide-binding and leucine-rich repeat receptors, disease resistance genes, broad-spectrum resistance

## Abstract

Powdery mildew (PM) is one of the most common *Cannabis sativa* diseases. In spite of this, very few documented studies have characterized the resistance genes involved in PM defense mechanisms, or sources of natural genetic resistance in cannabis. The focus of the present work is on the two primary mechanisms for qualitative resistance against PM. The first is based on resistance (*R*) genes characterized by conserved nucleotide-binding site and/or leucine-rich repeat domains (NLRs). The second one involves susceptibility (*S*) genes, and particularly mildew resistance locus o (*MLO*) genes, whose loss-of-function mutations seem to be a reliable way to protect plants from PM infection. Cannabis defenses against PM are thus discussed, mainly detailing the strategies based on these two mechanisms. Emerging studies about this research topic are also reported and, based on the most significant results, a potential PM resistance model in cannabis plant–pathogen interactions is proposed. Finally, innovative approaches, based on the pyramiding of multiple *R* genes, as well as on genetic engineering and genome editing methods knocking out *S* genes, are discussed, to obtain durable PM-resistant cannabis cultivars with a broad-spectrum resistance range.

## 1. Introduction

Plant diseases caused by pathogenic fungi, oomycetes, bacteria and viruses lead to yield losses, reducing their quality and economic value. These losses can be heavy; for instance, they can reach ~40% in rice and maize [1]. 

Powdery mildew (PM) is one of the most common plant diseases, caused by several fungi taxa belonging to the Erysiphales order of the Ascomycota phylum, which infects a wide range of plant species [2,3].

In contrast to well-known mycelial fungal/oomycete root rot pathogens, like *Fusarium* or *Pythium*, these biotrophic plant pathogens only infect plant tissues growing out of the ground, and the lower leaves are generally the most affected, with only their epidermal cell layer targeted [3]. In a susceptible host plant, the fungal conidium germinates, penetrates the cell wall and establishes a specialized structure, referred to as ‘haustorium’, to absorb nutrients [4]. Then, surface hyphae develop, as well as reproductive structures and new spores, resulting into an extensive surficial hyphal network. As the disease progresses, the PM may spread up and down the length of the crop. 

PM fungi grow well with high humidity levels and a moderate temperature, thus greenhouses conditions provide an ideal temperate environment for the spread of the infection, representing a great issue in breeding programs [5]. The disease also has a significant impact on plant growth and yield quality. For instance, a reduction of up to 25% in grain yield has been observed in susceptible wheat cultivars [6]. 

Asexual reproduction is the predominant strategy to generate PM fungi. The lifestyle of these organisms is a relevant issue for molecular investigations; in fact, efforts to establish a reliable protocol for the stable transformation of PM fungi have often been hampered by the difficulty to cultivate them in vitro [3], and many aspects of their biology have not been completely elucidated. However, several PM fungi genomes have been sequenced, for instance, those associated with barley, wheat, pea and *Arabidopsis* hosts [3].

*Cannabis sativa* belongs to the Cannabaceae family and is a dicotyledonous plant which is increasingly cultivated all over the world, due to its adaptability to a wide range of environmental conditions [7]. It is used as a source of industrial fiber, seed oil and food, as well as for health and recreational purposes [8]. The increase in cannabis breeding has led to a massive pathogen exposure, resulting in diseases playing a crucial role in its production. In spite of the availability of its genome sequences, few research works have investigated the pathogen defense mechanisms from a molecular point of view, as well as the underlying genetic and metabolic pathways [9]. 

Cannabis is susceptible to PM disease [10], which can reduce its yield and photosynthesis rate by damaging foliage and preventing the light from reaching its surface, resulting in premature plant senescence. PM represents a relevant limitation for cannabis production [11,12], and the economic impact of this disease has not yet been precisely evaluated in this crop [13].

The use of pesticides against PM in cannabis could have health risks for the consumer, and alternative methods include environmental control and applications of rhizobacteria promoting plant growth [14,15]. Currently, several products to manage PM in cannabis are available, like the bio-fungicide Regalia Maxx (an extract of giant knotweed) [15] and lacto-fermented products [15], such as Cyclone. Despite these pest management strategies, PM is still one of the most relevant biological diseases for cannabis, and the discovery and characterization of PM resistance genes is crucial for improving the cannabis industry in a sustainable way [10].

Resistance PM genes were found in hops (*Humulus lupulus*), the most closely related species to *C. sativa* [16,17]. In cannabis, despite a wide range of diseases being reported, very few documented *R* genes are known [9]. Emerging molecular studies have reported two primary mechanisms for qualitative resistance against PM in cannabis, but only recently: gene-for-gene resistance [18] and *mlo*-based resistance [13,19].

The first mechanism occurs when a pathogen-secreted effector protein is recognized by the compatible protein generated by the plant host resistance (*R*) genes, which are often characterized by conserved nucleotide-binding site (NBS) and/or leucine-rich repeat (LRR) domains (also termed NLRs) [20,21,22]. NLRs, whose mechanisms have been increasingly understood in recent years, are immune receptors and key components of the plant innate immune system, on which plants rely for defense against pathogen infections [23]. They represent the major class of intracellular innate immune receptors and the most represented group of resistance genes. To date, several NBS–LRR resistance genes and quantitative trait loci (QTLs) for plant resistance to pathogens were mapped in plants, some of which were also cloned [24,25], and, in many cases, a co-localization between QTLs and genes was highlighted. This made it possible to identify candidate genes and to develop molecular markers for plant resistance [24,25]. In cannabis, the involvement of NLRs in gene-for-gene interaction with PM has been recently demonstrated [18]. 

The second mechanism involves loss-of-function mutations of susceptibility (S) genes. The Mildew resistance locus o (*MLO*) genes are a family of *S* genes encoding seven transmembrane domain proteins only found in plants, thus helping the infection spread when interacting with PM fungi [26,27]. Their overexpression results in an enhanced susceptibility to PM [28]. Conversely, their loss-of-function mutations (*mlo)* seem to be a reliable way to protect plants from the infection, and they have a greater potential for durable PM resistance than R-gene resistance, which can be overcome more easily by new pathogen races [29]. Furthermore, *mlo*-based resistance is commonly non-race-specific and, as a consequence, is effective against the vast majority of PM isolates [30]. *mlo*-based resistance was initially observed in barley [31], and subsequently many researchers focused their efforts on understanding the molecular mechanisms behind it, discovering the broad-spectrum resistance (BSR) peculiarity in barley, and extending their research to other plant species [32]. 

In this work, we discuss the primary mechanisms for qualitative resistance against PM in cannabis, based on NLRs and *mlo*-based resistance. Emerging cannabis studies about both are reported and, taking into account the most significant results, innovative strategies based on the pyramiding of multiple *R* genes, as well as on genetic engineering and genome editing approaches, are discussed, to obtain durable PM resistant cannabis cultivars with a broad disease resistance spectrum. A potential PM resistance model, including NLR- and *mlo*-based resistance mechanisms in cannabis plant–pathogen interactions, is also proposed.

## 2. Broad-Spectrum Disease Resistance and NLR- and *mlo*-Based Mechanisms 

BSR confers resistance against more than one pathogen species (species-nonspecific) or against most races belonging to the same species (race-nonspecific) [33,34]. It is usually durable, remaining effective for long periods, even though the plant is exposed to the pathogen while still growing [33,34]. 

Most *R* genes are able to confer high levels of race-specific resistance against a single pathogen, even though some genes, such as those belonging to the wall-associated kinase (WAK) family, were found to be non-race-specific broad spectrum resistance genes [35]. However, due to mutations and virulence variations in pathogens, the effectiveness of the *R* genes is generally not very durable [34]. Conversely, the partial resistance regulated by QTLs is commonly race-nonspecific, although, in most cases, it provides an insufficient defense against pathogen attacks [34]. Combining *R* genes and QTLs is an effective strategy for disease control but may be technically challenging and requires a lot of time [34]. 

Given the above, BSR is a desirable trait and the selection of new cultivars with BSR characteristics has become a crucial crop breeding aim. 

Most BSR genes have been reported to encode pattern recognition receptors (PRRs), as well as defense-signaling and pathogenesis-related proteins (PRs) [34]. NLR proteins also mediate defense mechanisms against broad spectrum of pathogens [34,36,37,38], even though they may become ineffective due to virulence variations in pathogens. 

Furthermore, several *S* genes, whose loss-of-function mutations decrease the compatibility between pathogens and plant hosts, have been investigated and identified as BSR genes [34].

In the next sections our focus will be only on the two primary cannabis resistance mechanisms against PM: NLR- and *mlo*-based resistance mechanisms.

### 2.1. Nucleotide-Binding and Leucine-Rich Repeat Receptors and Their Role in the Immune System

The plant innate immune system consists of two layers: the first one includes the recognition of pathogen-associated molecular patterns (PAMPs) by membrane-associated PRRs, which activate PAMP-triggered immunity (PTI) [39,40]. The second layer results from the recognition of pathogen avirulence (Avr) effectors, leading to an effective and race-specific effector-triggered immunity (ETI), which is generally able to control specific pathogen attacks [20,23]. The ETI response mainly involves the nucleotide-binding and leucine-rich repeat receptors (NLRs) and other cytoplasmic proteins [36,38,41]. Both PRR and NLR-triggered immunity (NTI) lead to a downstream defense response, including the production of reactive oxygen species (ROS), a flux of extracellular calcium, kinase activation and transcriptional regulation in order to combat the infection [37,42]. ROS generation in response to the perception of the pathogen typically culminates in a hypersensitive response (HR) in many resistant genotypes, resulting in localized and very rapid cell death at the infection site [43]. Several transcription factor families, such as AP2/ERF, bHLH, MYB, NAC, WRKY and bZIP [44,45], can be involved in this immune response. After the immune recognition, defense signaling propagates to tissues distant from those where the infection occurred. Defense intensity and duration can be different between PTI and NTI [46]. NLRs induce a stronger and longer defense response over time, which often leads to a programmed cell death [21,37]. 

NLRs consist of a central NB domain, including the conserved P-loop motif required for ATP/ADP binding and NLR activity [47], and a C-terminal LRR, which is highly polymorphic and confers NLR recognition specificity [48]. NLRs are classified into two subgroups, according to their N-terminal domain: TIR-NB-LRR (TNL) and CC-NB-LRR (CNL) proteins, characterized by a Toll-like and a coiled-coil domain, respectively [24]. 

NLRs can be located in different subcellular organelles and districts, such as the cytoplasm, nucleus, plasma membrane and endoplasmic reticulum [37,49]. In plant genomes, they can be found either as isolated genes or organized in clusters, enabling the evolution of immune receptors [20,49]. More specifically, many NLRs, named sensor NLRs, perceive pathogen effectors, while others, referred to as helper NLRs, assist immune signaling [21]. NLRs can also be organized in networks, in which several helper NLRs act as signaling hubs for sensor NLRs and other immune receptors, which are localized on the plant cell surface. Pathogens primarily attempt to suppress NLR networks, facilitating the spread of the infection; thus, a deep understanding of the network interaction mechanisms could help to prevent plant disease [21]. 

NLRs were found to confer disease resistance against PM in many plant species. For instance, the mildew locus a (*Mla*) NLR gene has been demonstrated to be responsible for resistance against diverse fungal pathogens in cereal crops. In barley, *Mla* locus confers specific isolate immunity against the PM fungus *Blumeria graminis f.* sp. *hordei* (*Bgh*), and it has been proved that LRRs are largely responsible for the recognition specificity of structurally related effectors by MLAs [50], suggesting that MLA receptors may be driven in the *Bgh* recognition effectors by the presence of a common structural effector scaffold [50].

Regarding BSR genes encoding NLRs, the first identified species-nonspecific BSR NLR proteins were found in *Arabidopsis* resistance against two bacteria, *Ralstonia solanacearum* and *Pseudomonas syringae*, working synergically as a dual *R*-gene system [51]. Recently it was demonstrated in *Nicotiana benthamiana* that NLR proteins recognize the effectors of *Pseudomonas* and *Xanthomonas* species [52]. 

NLR-based resistance mechanisms have been the subject of several investigations to date [21].

### 2.2. mlo-Based Resistance

*mlo*-based resistance, initially detected as a natural mutation in an Ethiopian barley cultivar, was successfully introduced in Europe in agricultural programs conferring a broad-spectrum resistance against PM in barley [53,54]. Inactivation of barley MLO protein leads to an enhanced hydrogen peroxide accumulation in the epidermal cells and to cell death in the mesophyll, preventing *Bgh* penetration [55].

Recently, the barley *MLO* gene has been cloned, and its resistance mechanisms seem to include callose deposition, increased size of plant papilla and cell wall strengthening [56]. Now, more than half of spring barley is largely immune to PM, due to the introgression of *mlo* resistance into a broad panel of varieties [57]. Furthermore, researchers found that *mlo*-based resistance is also a feature of the dicotyledonous *Arabidopsis thaliana* [58] and many other plant species, such as cucumber [59], tobacco [60], apple [61], pea [62,63] and tomato [64]. *mlo*-based resistance mechanisms are generally different among plant species. In peas, two recessively inherited genes (*er1* and *er2*), representing the major natural sources of resistance against PM, are both responsible for a defense mechanism independent from HR and associated with the early interruption of pathogenesis after the differentiation of fungal appressoria [62,63]. In tomatoes, the loss-of-function of the *MLO* gene *SlMLO1* leads to a particular form of PM resistance, called *ol-2*, almost completely preventing pathogen penetration through the apposition of papillae at plant–pathogen interaction sites [64]. This resistance is caused by a natural polymorphism, resulting in a small deletion within the *MLO* coding region.

To date, *mlo* resistance has been found as a natural mutation in several crops or produced through induced mutagenesis, gene silencing or gene knock-out [29].

Structural and functional analyses of MLO proteins revealed that the conserved calmodulin-binding domain (CaMBD) seems to be required for full susceptibility to PM infection in barley [65]. 

Moreover, MLO proteins are characterized by four conserved cysteines [66], and novel conserved peptide domains have been discovered [67]. However, to the best of our knowledge, little is known about the molecular function and biochemical activity of these proteins. 

*MLO* genes are found in many crop species, including angiosperms, gymnosperms, lycophytes, bryophytes, algae and other unicellular eukaryotes [19], suggesting that MLO is an ancient eukaryotic protein. To date, a total of ~200 *MLO* genes have been identified, which are characterized by rich nucleotide diversity and only partially containing a CaMBD [68]. 

*MLO* genes encode plant-specific proteins sorted in seven conserved clades, according to the most common classification [29], with IV and V clades appearing to be associated with MLO proteins involved in PM susceptibility in monocots and dicots, respectively [29,69]. 

Although *mlo*-based resistance genes have been investigated in several monocot and dicot species, they have been poorly studied in cannabis, as well as other genes involved in disease defense mechanisms [9]. However, in recent years, investigations about *MLO* genes revealed many key features and characteristics of this family in cannabis, such as the presence of seven transmembrane domains, the presence of the MLO functional domain and the presence of all seven clades, similarly to other crops [19].

Furthermore, to date and to the best of our knowledge, barley (*Hordeum vulgare*) *mlo* genes are the only race-nonspecific BSR *mlo* genes identified [31], but their effective and durable resistance has encouraged the identification and characterization of many other *MLO* orthologs in several plant species, such as *Arabidopsis AtMLO2*, *AtMLO6* and *AtMLO12* [58] and cucumber *CsaMLO8* [59], in addition to the already mentioned tomato *SlMLO1* [64] and pea *Er1/PsMLO1* PM [62,63].

## 3. Powdery Mildew Resistance in Cannabis

Cannabis plants are susceptible to the predominant PM pathogen (*Golovinomyces* spp.) [10,11,70,71]. Symptoms initially appear as white circular patches of ectophytic mycelia and conidia on the cannabis leaf surface, which later cover the entire surface, and then flowers and buds [10]. 

*Golovinomyces* species were found to be a strong post-harvest contaminant of cannabis [18]. These species are *G. ambrosiae, G. spadiceus and G. cichoracearum* [11,72], whose morphological characters overlapped with several *Golovinomyces* spp. Furthermore, according to a recent *Golovinomyces* taxonomic revision based on a multi-locus phylogenetic examination, *G. ambrosiae* and *G. spadiceus* were found to form a single undifferentiated clade [73]. 

In spite of the fact that the vast majority of PM infections in cannabis come from *Golovinomyces*, another fungal species has been showed to infect this crop, the *Podosphaera macularis*, which commonly targets hop plants [74,75]. Interestingly, a host-resistance response to this species was observed in ‘TJ’s CBD’, a cannabis cultivar susceptible to *G. ambrosiae* [76]. This suggests that, in this cultivar, an *R* gene conferring resistance to *P. macularis* may be found. Symptoms are evident on foliage, but they are mainly localized on inflorescences in the lower portions of the plant [74]. In greenhouse environments, *G. ambrosiae* was the most common PM pathogen, while *P. macularis* was found in plants located in the fields [75]. To date, the *P. macularis* ability to expand to other sites is still not known [75].

In a recent study [19], *CsMLO* genes were characterized and their role in PM susceptibility as negative regulatory factors in the cannabis immune system was underlined. Here, the analysis was carried out using the genomes of the ‘Purple Kush’ and ‘Finola’ cannabis cultivars [77], of ‘CBDRx’ [78] and of female and male ‘Jamaican Lion’ [79]. The *CsMLO* genes study revealed particular amino acid positions, which are present in well-conserved regions, and the phylogenetic analysis of fifteen of them showed that, in all the considered genomes, seven distinct clades (I–VII) were present, as reported in other crops. The focus was on two genes of clade V, *CsMLO1* and *CsMLO4*, both associated with seven transmembrane domains. In fact, the expression analysis revealed that they are remarkably up-regulated during *G. ambrosiae* infection and were identified as candidates potentially involved in PM susceptibility. The study also included the analysis of amino acids within *CsMLO1* and *CsMLO4* genes in ~30 commercial cannabis cultivars, revealing several variations, which could influence their related protein functions. Furthermore, in the examined genomes, natural loss-of-function mutations in clade V MLOs were not observed, suggesting that a complete resistance to PM could be rare in commercial cannabis cultivars. Therefore, obtaining a resistant phenotype could be challenging, considering the recessive nature and the genetic redundancy of several *CsMLO* genes [19].

Another very recent study characterized a new source of PM resistance, confirming the crucial role of *MLO* genes in PM susceptibility in cannabis [13]. Here, the cannabis cultivar ‘FL 58’ was investigated. The choice of this cultivar was due to the fact that it was subjected to controlled PM inoculation for three consecutive years and no significant infection was observed, thus representing a potential source of PM resistance in *C. sativa* [80]. Furthermore, two populations, coming from the cross of ‘FL 58’ with the PM susceptible cultivar ‘TJ’s CBD’, were used to identify the genetic basis of PM resistance. These populations were genotyped with single nucleotide polymorphisms (SNPs) and a consensus genetic map was generated. Results showed at least five unique and never identified loci contributing to PM resistance/susceptibility variation. The most associated marker on chromosome 1 was located near the ‘FL 58’ *CsMLO1* gene, which was identified as the primary candidate *S* gene to PM, and it was found to be rare in the cannabis pangenome produced by the Michael lab [13]. Further analyses supported the hypothesis that PM resistance is the effect of the insertion identified in the ‘FL 58’ *CsMLO1* sequence, leading to irregular mRNA splicing, and resulting in a premature termination codon. Transcripts encoding a premature stop were found to be ~35 to 65 times more abundant than *CsMLO1* full-length transcripts. The consequent strong reduction in functional *CsMLO1* proteins could justify the resistance observed in ‘FL 58’ and in other homozygous genotypes [13].

Another significant work showed that the first *R* gene identified in cannabis was represented by a single dominant locus and was able to confer complete resistance to the PM pathogen *G. ambrosiae* [18]. Here, for PM pathogen identification, sequence data from 5.8S and 28S rDNA and ITS regions 1 and 2 were generated, and the results showed that the isolate shared 100% sequence homology with *G. spadiceus*/*G. ambrosiae* pathogens. The experiments carried out in this study, based on several cannabis cultivars, revealed resistant phenotypes, such as those found in the ‘PNW39’ population, where PM colonies are absent. Then, on the basis of the ‘CBDRx’ cannabis genome annotation, and while adopting the linkage mapping approach with ~10,000 SNP markers, ten candidate genes of a single dominant *R* gene, named *PM1*, were identified. This gene resulted in co-localization with the SNP markers LH3804, LH31156, and LH17304 on chromosome 2, and, in the area surrounding the LH3804 locus, a region containing NLRs was identified. More specifically, a cluster of putative disease resistance proteins contained N-terminal coiled-coil (CC) and nucleotide-binding arc (NB-ARC) domains, and two genes with LRR characteristics were detected. Three genes, annotated as tetratricopeptide repeat-containing proteins, were also observed. In conclusion, it can be stated that Mihalyov and Garfinkel’s study [18] provides crucial insights for further genetic cannabis PM resistance research, in order to improve its immunity system.

Furthermore, it is known that NLRs are involved in resistance to PM in several other plant species, like *Vitis vinifera* [81] and *Triticum aestivum* [82], and NBS proteins have been associated with candidate PM resistance genes in *Humulus lupulus* [17]. According to these results and Mihalyov and Garfinkel’s findings [18], NLR-based PM resistance may be hypothesized for cannabis. 

Thaumatin-like proteins (TLPs), whose antifungal properties are known [83], were found in hops PM (*Podospheara macularis*) resistance [84]; however, to the best of our knowledge, there is no evidence of this in cannabis.

**Figure 1:**
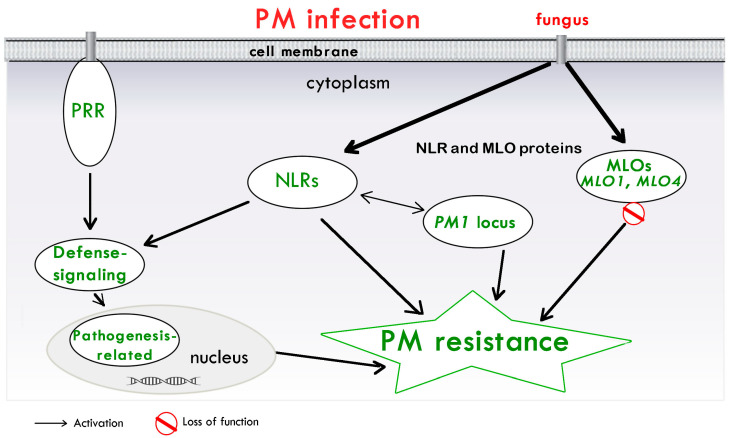
A model of the main mechanisms of PM resistance in cannabis. PAMPs are perceived by membrane-associated PRRs, which activate defense signaling. NLRs recognize pathogen-secreted proteins. These recognitions, in turn, activate immune signaling cascades, resulting in the synthesis of numerous pathogenesis-related proteins to confer PM resistance. Proteins encoded by *PM1* gene, represented by a single dominant locus and associated with a region containing NLRs, are shown. Proteins encoded by *MLO* genes (*MLO1* and *MLO4*), which can lead to PM cannabis resistance, are also included. Abbreviations: Mildew resistance locus o (*MLO*) gene; NLR, nucleotide-binding and leucine-rich repeat receptor; PM, powdery Mildew; PRR, pattern recognition receptor.

On the basis of the existing literature and the emerging studies about cannabis PM resistance, a schema of the involved mechanisms is illustrated in Figure 1.

## 4. Development of Durable Cannabis Cultivars Resistant to PM

Breeding resistant cultivars using resistance genes was the most effective and convenient method to control plant diseases [85,86]. However, the common loss of *R* gene resistance limits the use of single genes in innovative breeding approaches. Pyramiding resistance genes endowed with complementary pathogen resistance spectra has been successfully tested and is an effective strategy for achieving durable resistance. For instance, by using the marker-assisted selection (MAS) technique, resistance genes have been pyramided to generate new crop varieties resistant to several infections, including PM [34]. 

To date, only a single PM resistance locus in cannabis has been characterized [18]; thus, the identification and introgression of durable PM resistance into elite germplasm is a fundamental approach in developing effective pathogen management programs in cannabis.

Furthermore, high-throughput molecular marker investigations, like those of Stack et al. [13] for *CsMLO1* and Mihalyov and Garfinkel [18] for *PM1*, can provide a great starting point for the gene pyramiding approach to obtain resistant and durable cannabis cultivars; also, this process does not cause physical linkage breaking, since the *CsMLO1* gene is not linked to *PM1* on cannabis chromosome 2. 

The combination of pyramided *R* genes with multiple QTLs to achieve broad-spectrum resistance has also been investigated [85,86,87]; for instance, pyramiding *R* genes with QTLs has been proved to be effective in controlling stripe rust and PM in several spring wheat breeding programs [88]. 

On the other hand, omics and multi-omics approaches have also allowed the investigation of defense response pathways in many crops and have been broadly used in medicinal plants, identifying candidate resistance genes and leading to an in-depth knowledge of the underlying molecular mechanisms [89,90]. They could be a great starting point for genome editing/genetic engineering studies, in order to obtain disease-resistant cannabis varieties [90].

Genetic engineering methods to improve desirable traits in cannabis have been applied in very few investigations [91], and the functions of cannabis R genes are not fully validated yet. In fact, it is challenging to regenerate fully developed cannabis transgenic plants [92]. 

The first engineered cannabis line was obtained using an *Agrobacterium*-mediated transformation [93], and by applying this approach, the development of transgenic callus from cannabis was obtained [94].

Recently, advancements have been made in the engineering of sensitized NLR variants, with the final aim of recognizing a wider spectrum of effectors [34,95]. The diversity of NLRs, which are able to sense effectors directly or indirectly through other proteins, allowed researchers to apply several engineering methods to improve disease resistance in plants [21,96]. Studies about NLR mutations have been carried out for several years. Among them, one investigation, using a homology modelling approach, hypothesized that mutations increasing the sensitivity of the NLR protein are localized around the conserved ATP/ADP binding site, which mediates the NLR activation state [97]. Another study investigated mutations in the conserved coiled-coil and nucleotide-binding domains of these receptors to increase their response range [98]. More recent studies provided new insights aiding the design of bespoke NLRs [96,99,100], and others demonstrated an enhanced recognition of pathogen effectors by using NLR engineering methods [96,101,102].

Similar approaches could be used to improve cannabis PM disease resistance; for instance, the cannabis *R* gene *PM1*, which was found to be co-localized with SNP markers in a region containing NLRs, could be the subject of genetic engineering projects [18].

CRISPR/Cas9 (clustered regularly interspaced short palindromic repeats/CRISPR-associated protein 9) technology, which is still rarely used in cannabis, could be applied to modify gene regulation and increase pathogen resistance, as already undertaken in other recalcitrant plants, including grapes [103,104]. 

Editing technologies, including the CRISPR/Cas9 approach, have also made it possible to use the targeted mutagenesis of *S* genes in several important crops, with the aim of generating BSR cultivars. Transcription activator-like effector nucleases (TALEN) and CRISPR/Cas9 technologies were used to target the *MLO* loci in wheat in order to obtain PM resistant crops [105]. Knocking-out the *MLO* ortholog *SlMLO1* resulted in full resistance to the PM fungus in tomatoes [106]. CRISPR/Cas9-mediated mutagenesis of the *MLO3* gene provided an enhanced resistance to PM in grapevines [107], *MLO7* was used as a host susceptibility gene to improve grapevine and apple disease resistance to PM [108] and *mlo*-mediated resistance against *Podosphaera xanthii* was successfully used in cucumber [109]. Furthermore, a targeted deletion in the wheat MLO-B1 locus conferred robust PM without growth penalty and yields loss [110] 

These findings clearly show that the manipulation of *S* genes, such as *MLO* genes, is a powerful approach to generate pathogen resistance in important crops.

Editing technologies could be an efficient method to introduce *S* gene knockouts in cannabis and to promote hybrid cannabis cultivar development. For instance, in the previously discussed study of Pépin et al. [19], the two identified genes *CsMLO1* and *CsMLO4*, which are significantly involved in cannabis PM susceptibility, could be used for these purposes, and a double-knockout would be necessary to confer *mlo*-based resistance. Due to the presence of multiple copies of *CsMLO1* gene in the cannabis genome [19], multiple genes would need to be knocked out to confer PM resistance. Furthermore, according to the investigations of Stack et al. (2023) [13] and Pépin et al. (2021) [19], the knockout of *CsMLO1* and a predicted multi-genic model based on the gene expression analysis of both *CsMLO1* and *CsMLO4* genes could also be a great strategy for achieving complete cannabis PM resistance. 

However, further studies about *MLO* knockout approaches are necessary before applying them to cannabis; we also need to establish if the *mlo*-based resistance mechanism against *G. ambrosiae* is effective against *P. macularis* [13].

## 5. Conclusions

Few documented studies have characterized cannabis resistance genes involved in PM defense mechanisms, and even less have investigated genes for durable resistance. However, the most relevant works here reported [13,18,19], in our opinion, represent a great starting point for further research investigations in this field.

Gene editing, and particularly knocking out the identified cannabis *MLO* genes, as well as genetic engineering approaches aimed to enable cannabis NLR variants to recognize a wider spectrum of effectors, could be great strategies to obtain cannabis cultivars with durable and/or BSR resistance PM characteristics.

A deeper understanding of the underlying molecular mechanisms in which these genes and proteins are involved, as well as of cannabis PM fungi interaction, is leading to crucial innovations in the development of resistant cannabis cultivars. 

## Data Availability

Not applicable.

## Abbreviations

List of abbreviations used in this manuscript:

BSR	Broad-spectrum resistance
CRISPR/Cas 9	Clustered regularly interspaced short palindromic repeats/CRISPR-associated protein 9
ETI	Effector-triggered immunity
IGS	Inter generic spacer
ITS	Internal transcribed spacer
LRR	Leucine-rich repeat
MAS	Marker-assisted selection
Mlo	Mildew resistance locus o
NBS	Nucleotide-binding site
NTI	NLR-triggered immunity
PAMPs	Pathogen-associated molecular patterns
PCR	Polymerase chain reaction
PM	Powdery mildew
PRRs	Pattern recognition receptors
PTI	PAMP-triggered immunity
QTL	Quantitative trait loci
ROS	Reactive oxygen species
SNPs	Single nucleotide polymorphisms
TALEN	Transcription activator-like effector nucleases
TLPs	Thaumatin-like proteins
